# Is the sky the limit? On the expansion threshold of a species’ range

**DOI:** 10.1371/journal.pbio.2005372

**Published:** 2018-06-15

**Authors:** Jitka Polechová

**Affiliations:** 1 University of Vienna, Department of Mathematics, Vienna, Austria; 2 Institute for Science and Technology (IST Austria), Klosterneuburg, Austria; Estación Biológica de Doñana (EBD-CSIC), Spain

## Abstract

More than 100 years after Grigg’s influential analysis of species’ borders, the causes of limits to species’ ranges still represent a puzzle that has never been understood with clarity. The topic has become especially important recently as many scientists have become interested in the potential for species’ ranges to shift in response to climate change—and yet nearly all of those studies fail to recognise or incorporate evolutionary genetics in a way that relates to theoretical developments. I show that range margins can be understood based on just two measurable parameters: (i) the fitness cost of dispersal—a measure of environmental heterogeneity—and (ii) the strength of genetic drift, which reduces genetic diversity. Together, these two parameters define an ‘expansion threshold’: adaptation fails when genetic drift reduces genetic diversity below that required for adaptation to a heterogeneous environment. When the key parameters drop below this expansion threshold locally, a sharp range margin forms. When they drop below this threshold throughout the species’ range, adaptation collapses everywhere, resulting in either extinction or formation of a fragmented metapopulation. Because the effects of dispersal differ fundamentally with dimension, the second parameter—the strength of genetic drift—is qualitatively different compared to a linear habitat. In two-dimensional habitats, genetic drift becomes effectively independent of selection. It decreases with ‘neighbourhood size’—the number of individuals accessible by dispersal within one generation. Moreover, in contrast to earlier predictions, which neglected evolution of genetic variance and/or stochasticity in two dimensions, dispersal into small marginal populations aids adaptation. This is because the reduction of both genetic and demographic stochasticity has a stronger effect than the cost of dispersal through increased maladaptation. The expansion threshold thus provides a novel, theoretically justified, and testable prediction for formation of the range margin and collapse of the species’ range.

## Author summary

The flow of genetic diversity across environments has conflicting effects. On the beneficial side, it increases the genetic variation that is necessary for adaptation and counters the loss of genetic diversity due to genetic drift. However, it may also swamp adaptation to local conditions. This interplay is crucial for the expansion of a species’ range. In this work, I develop a theory that shows how range expansion depends on two dimensionless parameters: (i) the fitness cost of dispersal—a measure of environmental heterogeneity—and (ii) the strength of genetic drift—a measure of the reduction of genetic diversity. The more heterogeneous an environment, the more challenging it is to expand into, and the lower the genetic diversity, the more limited is the scope for potential adaptation. Together, these two parameters define an ‘expansion threshold’: adaptation fails when the number of individuals accessible by dispersal within one generation is so small that genetic drift reduces genetic diversity below that required for adaptation to a heterogeneous environment. This threshold provides a novel, theoretically justified, and testable prediction for the formation of a range margin and for the collapse of a species’ range in two-dimensional habitats.

## Introduction

Species’ borders are not just determined by the limits of their ecological niche [1, 2]. A species’ edge is typically sharper than would be implied by continuous change in the species’ environment (reviewed in [3, Table 2]). Moreover, although species’ ranges are inherently dynamic, it is puzzling that they typically expand rather slowly [4]. The usual—but tautological—explanation is that lack of genetic variation at the range margin prevents further expansion [5]. Indeed, a species’ range edge is often associated with lower neutral genetic variation [3, 6–11], suggesting that adaptive genetic variation may be depleted as well [12]. Yet why would selection for new variants near the edge of the range not increase adaptive genetic variance, thereby enabling it to continuously expand [5, 13]? Haldane [14] proposed a general explanation: even if environmental conditions vary smoothly, ‘swamping’ by gene flow from central to marginal habitats will cause more severe maladaptation in marginal habitats, further reducing their population density. This would lead to a sharp edge to a species’ range, even if genetic variance at the range margin is large. However, the consequences of dispersal and gene flow for evolution of a species’ range continue to be debated [15–18]: a number of studies suggest that intermediate dispersal may be optimal [19–23]. Gene flow across heterogeneous environments can be beneficial because the increase of genetic variance allows the population to adapt in response to selection [13]. Current theory identifies that local population dynamics, dispersal, and evolution of niche-limiting traits (including their variance) and both genetic and demographic stochasticity are all important for species’ range dynamics [13, 19–21, 24–28]. Yet these core aspects have not been incorporated into a single study that would provide testable predictions for range limits in two-dimensional habitats.

**Fig 1:**
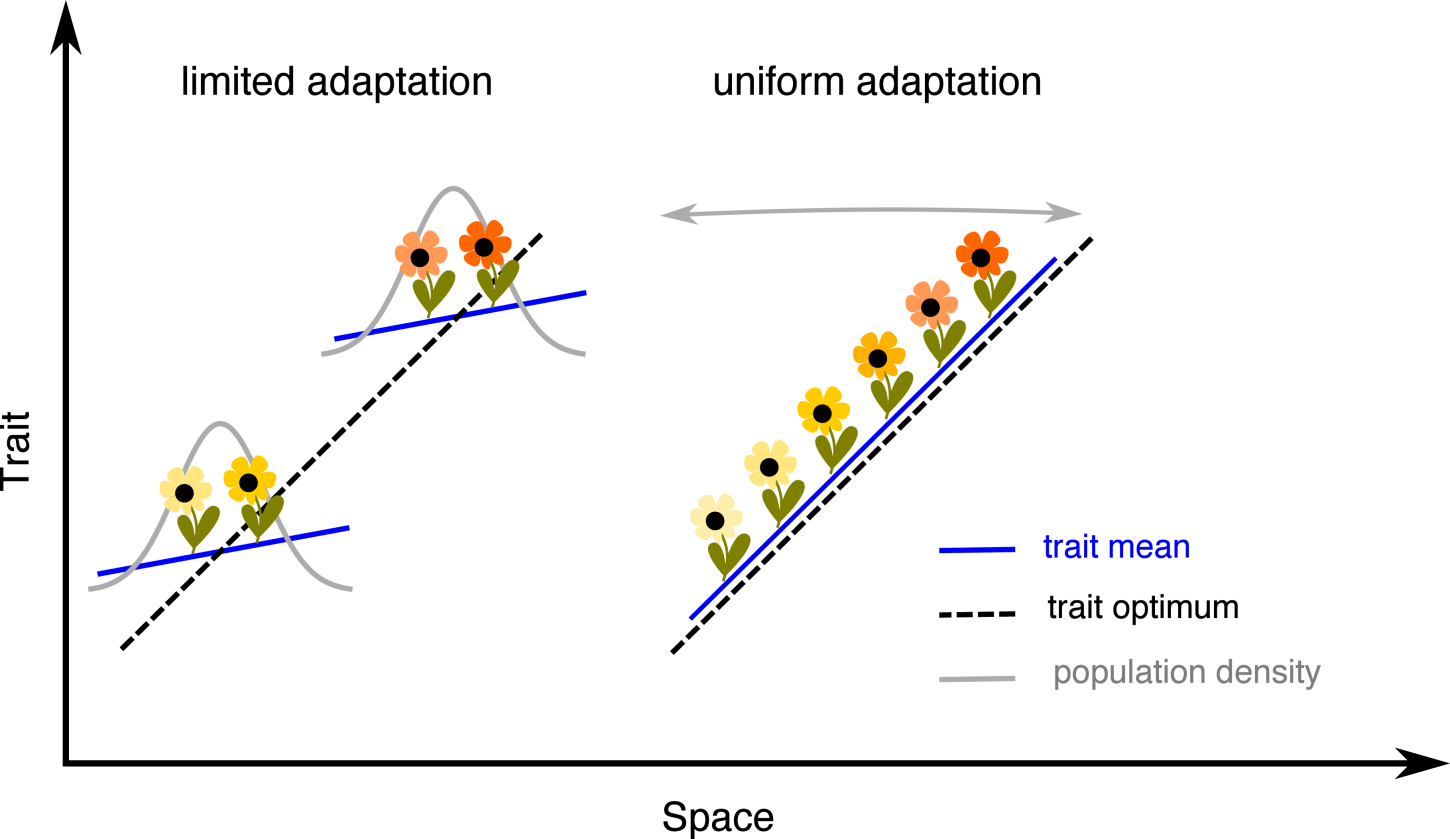
Two modes of adaptation. Assuming that genetic variance is fixed, deterministic theory [24] predicts that there are two modes of adaptation to an environmental gradient. When the effective environmental gradient *B* is steep relative to the genetic potential for adaptation *A*, clinal adaptation fails, and the population only matches the optimum at the very centre of its range (limited adaptation). These parameters can be understood as fitness loads scaled relative to the strength of density dependence r* (see [24, 30] and [31, Appendix D]). *A* is a measure of standing load due to genetic variance Ar*, and *B* is a measure of dispersal load *B*^2^r*^2^—the maladaptation incurred by dispersal across heterogeneous environment. Thus, conversely, when the standing load is large relative to the dispersal load *A*>*B*^2^/2, a population adapts continuously, gradually expanding its range (uniform adaptation). Black dashed lines depict the trait optimum; blue lines depict the trait mean. Population density is shown in grey: it has a sharp and stable margin for limited adaptation, but it is steadily expanding under uniform adaptation. Two subpopulations (or perhaps species) are given for illustration of limited adaptation—depending on further parameters and initial conditions (discussed in this study), a wide species’ range with uniform adaptation can collapse to a single population or fragment to multiple subpopulations.

As Haldane [14] previously pointed out, it is important to consider population and evolutionary dynamics across a species’ range jointly, as their effects interact. Due to maladaptation, both the carrying capacity of the habitat and the population growth rate are likely to decrease—such selection is called ‘hard’ [29]. Classic deterministic theory [24] shows that when genetic variance is fixed, there are two stable regimes of adaptation to a spatially varying optimum (see Fig 1): (i) a ‘limited adaptation’, in which a population is only adapted to a single optimum or becomes a patchy conglomerate of discrete phenotypes, or (ii) continuous or ‘uniform’ adaptation, which is stable when the genetic variance, measured in terms of its cost in fitness (standing genetic load), is large relative to the maladaptation incurred by dispersal between environments (dispersal load). Under uniform adaptation, a species’ range gradually expands—a stable boundary only forms when the genetic variance is too small to allow continuous adaptation to the spatially variable environment, and hence, limited adaptation is stable.

When genetic variance can evolve, such a limit no longer exists in infinitely large populations: the population maintains continuous adaptation as the environmental gradient steepens [13]. Deterministic theory thus predicts that a sharp and stable boundary to a species’ range does not form when the environment changes smoothly. Uniform adaptation is the only stable regime when genetic variance can freely evolve in the absence of genetic drift [13], yet there is a limit to the steepness of the gradient. This limit arises because both the standing genetic load and the dispersal load increase as the gradient steepens, reducing the mean fitness (growth rate) of the population: when the mean fitness approaches zero, the population becomes extinct. Obviously, ignoring genetic drift is then unrealistic. In finite populations, genetic drift reduces local genetic variance [32], potentially qualitatively changing the dynamics. Indeed, it has been shown that for one-dimensional habitats (such as rivers), a sharp range margin arises when the fitness cost of dispersal across environments becomes too large relative to the efficacy of selection versus genetic drift [26]. However, most species live in two-dimensional habitats. There, allele frequencies can fluctuate over a local scale, as the correlations between them decline much faster across space than they do in linear habitats [33], and the effect of genetic drift changes qualitatively, becoming only weakly dependent on selection [34]. Is there still an intrinsic threshold to range expansion in finite populations when dispersal and gene flow occur over two-dimensional space rather than along a line? If so, what is its biological interpretation?

## Results

I study the problem of intrinsic limits to adaptation in a two-dimensional habitat. Throughout, I assume that the species’ niche is limited by stabilising selection on a composite phenotypic trait. This optimum varies across one dimension of the two-dimensional habitat—such as temperature and humidity with altitude. Demography and evolution are considered together. Selection is ‘hard’: both the rate of density-dependent population growth and the attainable equilibrium density decrease with increasing maladaptation. Both trait mean and genetic variance can freely evolve via change in allele frequencies and the associations among them (linkage disequilibria). The populations are finite, and both genetic and demographic stochasticity are included. The model is first outlined at a population level in terms of coupled stochastic differential equations. While it is not possible to obtain analytical solutions to this model, this formalisation allows us to identify the effective dimensionless parameters that describe the dynamics. Next, individual-based simulations are used to determine the driving relationship between the key parameters and test its robustness. The details are described in the Model section of the Methods.

The dynamics of the evolution of a species’ range, as formalised by this model, are well described by three dimensionless parameters, which give a full description of the system. The first dimensionless parameter carries over from the phenotypic model [24]: the effective environmental gradient *B* measures the steepness of the environmental gradient in terms of maladaptation incurred by dispersal across a heterogeneous environment. The second parameter is the neighbourhood size of the population, 𝒩, which can be understood as the number of diploid individuals within one generation’s dispersal range. Originally, neighbourhood size was defined by Wright [35] as the size of the single panmictic diploid population that would give the same probability of identity by descent in the previous generation. The inverse of neighbourhood size 1/ 𝒩 hence describes the local increase of homozygosity due to genetic drift. The third dimensionless parameter is the ratio s/r* of the strength of selection *s* per locus relative to the strength of density dependence, r*. Detailed description of the parameters and their rescaling can be found in the Methods sections Parameters and Continuous model: Rescaling.

**Fig 2:**
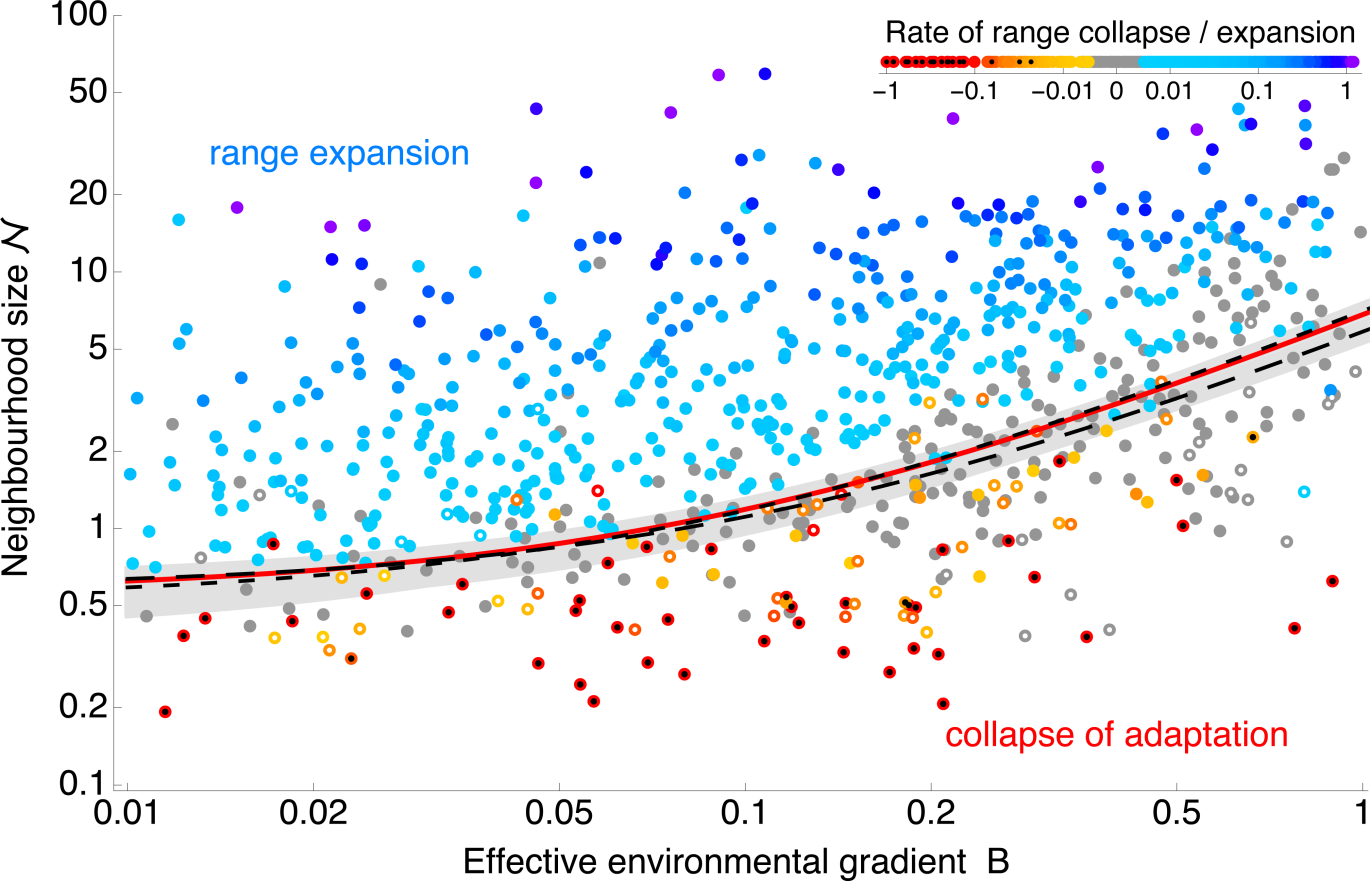
Two dimensionless parameters—the neighbourhood size 𝒩 and the effective environmental gradient B —give a clear prediction whether a species’ range can expand. The red line shows the fitted boundary between expanding populations (in blue) and collapsing ranges (red hues): populations expand above the expansion threshold when 𝒩 ⪆ 6.3*B* + 0.56. The grey region gives 95% bootstrap confidence intervals, whilst the dashed lines depict the predicted expansion threshold for weak selection, *s*/r*<0.005 (− −), and for strong selection, *s*/r*>0.005 (— —). Stagnant populations, changing by less than 5 demes per 1,000 generations, are shown in grey. Solid (blue, grey) dots depict populations with uniform adaptation (illustrated by Fig 3). Open circles denote populations in which continuous adaptation has collapsed and the population consists of many discrete phenotypes adapted to a single optimum each (limited adaptation, Fig 4), whilst local genetic variance is very small. (Specifically, these are defined by mean heterozygosity smaller than 10% of the predicted value in the absence of genetic drift.) Simulations were run for 5,000 generations, starting from a population adapted to a linearly changing optimum in the central part of the available habitat. Populations that went extinct are marked with a black dot. Note that both axes are on a log scale. The top corner legend gives the colour-coding for the rate of range collapse and expansion in units of demes per generation; rates of collapse are capped at −1. The expansion threshold is fitted as a step function changing linearly along *B*: all blue dots are assigned a value of 1; all red dots and open circles are assigned a value of 0. The expansion threshold has a coefficient of determination R^2^ = 0.94, calculated from 589 simulations (all but well-adapted stagnant populations). Data for this figure—and all subsequent ones—are deposited at Dryad Digital Repository, https://doi.org/10.5061/dryad.5vv37 [36].

In order to see how the rescaled parameters capture the evolution of a species’ range, I simulated 780 evolving populations, each based on different parameterisations, adapting to a linear gradient in the optimum. Depending on the parameters, the population either expands, gradually extending its phenotypic range by consecutive sweeps of loci advantageous at the edges, or the species’ range contracts or disintegrates as adaptation fails. Fig 2 shows the results of the projection from a 10-dimensional parameter space of the individual-based model (see Methods sections Individual-based simulations and Parameters) into a two-dimensional space. The axes of Fig 2 represent the first two compound dimensionless parameters: (i) the effective environmental gradient *B* and (ii) the inverse of neighbourhood size 1/ 𝒩, which describes the effect of genetic drift on the allele frequencies.

**Fig 3:**
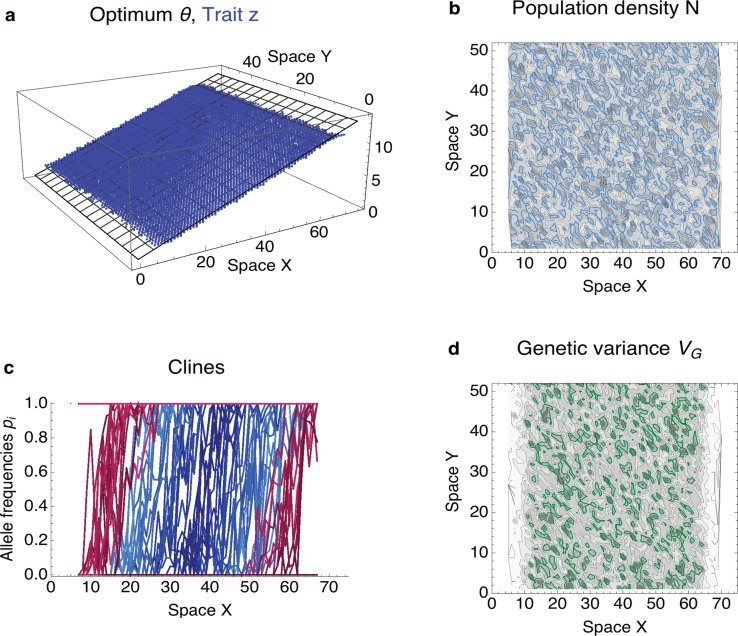
Uniform adaptation: Above the expansion threshold, the population expands gradually through the available habitat. (**a**) Trait (in blue) closely matches the environmental gradient (grey) along the x-axis. (**b**) Population steadily expands, whilst population density stays continuous across space, with $\bar{N}=19\pm 5.8$ (mean ± standard deviation). The prediction at $\hat{N}=20$ is shown by the blue contours; darker shading represents higher density. (**c**) Adaptation to the environmental gradient is maintained by a series of staggered clines: as each allele frequency changes from 0 to 1, the trait value increases by *α*. Population starts from the centre (blue hues reflect initial cline position relative to the centre of the range), and as it expands, new clines arising from loci previously fixed to 0 or 1 contribute to the adaptation (in red hues). At each location, multiple clines contribute to the trait (and variance); clines are shown at *Y* = 25. (**d**) Genetic variance changes continuously across space with mean ${\bar{V}}_{G}=0.032\pm 0.017$ and stays slightly lower than is the deterministic prediction (green contours, *V*_G_ = 0.045; higher variance is illustrated by darker shading). Deterministic predictions are based on [13] and are explained in the Methods section, along with the specification of the unscaled parameters. The population evolves for 2,000 generations, starting from a population adapted to the central habitat. The predicted neighbourhood size is $\hat{N}=34.6$; effective environmental gradient is *B* = 0.48.

These two dimensionless parameters *B* and 𝒩 give a clear separation between expanding populations, in which the neighbourhood size 𝒩 is large relative to the effective environmental gradient *B* (shown in blue, Fig 2), and the rest, in which adaptation is failing. The separation gives an ‘expansion threshold’, estimated at 𝒩 ≈ 6.3*B* + 0.56 (red line). Above the expansion threshold, populations are predicted to expand (see Fig 3); below it, adaptation fails abruptly. If conditions change uniformly across space (as in these simulation runs, with constant gradient and carrying capacity), this means that adaptation fails everywhere—a species’ range then either collapses from the margins (Fig 2, red hues) and/or disintegrates (Fig 2, open circles), forming a fragmented metapopulation (i.e., a spatially structured population consisting of discrete locally adapted subpopulations with limited dispersal among them).

**Fig 4:**
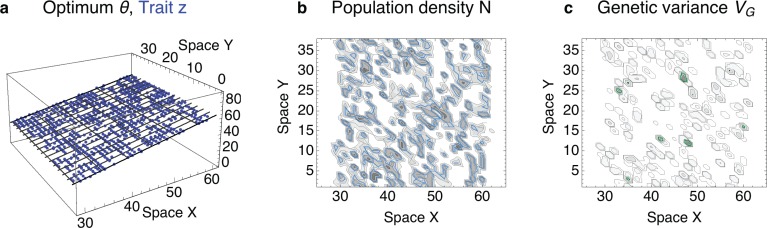
A metapopulation can form when the population is below the expansion threshold throughout its range. The population fragments rapidly (within tens of generations) to small patches of tens to a few hundred individuals whilst losing local adaptive variation. In two-dimensional habitats, such a metapopulation with limited adaptation can persist for a long time. Nevertheless, the population very slowly contracts, eventually forming a narrow band adapted to a single optimum. (**a**) The distribution of phenotypes across space is fragmented. (**b**) The subpopulations are transient, although they are stabilised by dispersal across space, especially along the neutral direction with no change in the optimum (Y). Locally, the population density may be higher than under uniform adaptation; blue contours depict the deterministic prediction for population density under uniform adaptation, N = 3. The realised density is about $\bar{N}=3.05\pm 1.7$ (standard deviation); darker shading represents higher density. (**c**) The adaptive genetic variance is low on average ($\bar{V_{G}}=0.02\pm 0.06$)—about an order of magnitude lower than would be maintained by gene flow under uniform adaptation (shown in green contours, *V*_G_ = 0.23). Typically, only a few clines in allele frequencies contribute to the genetic variance within a subpopulation. The parameterisation and predictions are detailed in the Individual-based simulations section of the Methods; predicted neighbourhood size is $\hat{N}=2.7$, effective environmental gradient is *B* = 0.48. Shown here after 5000 generations—the population collapses to a narrow band (at *X* = 45) after a further 20,000 generations and then appears persistent.

When a metapopulation forms, it exhibits an extinction and colonisation dynamics. The subpopulations drift freely along the neutral spatial axis. Because the trait distributions of the subpopulations are unstable, the subpopulations also drift slowly along the environmental gradient. Over time, the metapopulation very slowly collapses to a virtually single trait value, with many subpopulaitons along the neutral axis. The subpopulations forming this metapopulation have only a very narrow phenotypic range and maintain locally only minimal adaptive variance. They correspond to the limited adaptation regime identified for a phenotypic model with genetic variance as a parameter [24]. In contrast to one-dimensional habitats [26], these patchy metapopulations are stabilised by dispersal from surrounding subpopulations in the two-dimensional habitat and can thus persist for a long time. An example of such a metapopulation is given in Fig 4.

Interestingly, the third dimensionless parameter *s*/r* has no detectable effect on the form of the expansion threshold. In other words, whilst the expansion threshold reflects the total fitness cost of dispersal in a heterogeneous environment, it appears independent of the strength of selection per locus *s*: the dashed lines in Fig 2 compare the estimated expansion threshold for small and large *s*/r*. Increasing the strength of selection is inefficient in aiding drift-limited adaptation, in line with the expectation that the effect of genetic drift is only very weakly dependent on selection in two-dimensional habitats [27](see also S1 Fig). This suggests that genetic basis of adaptation is not important for a drift-induced limit to a species’ range. Yet it is plausible that there is another limit, in which selection per locus becomes important [27], that arises when the optimum changes abruptly and even when the population (neighbourhood) size is large (i.e., in an entirely different regime). A dedicated synthesis connecting the step-limited and drift-limited regimes would be of a clear interest. Importantly, once genetic drift starts to have an effect, the habitat needs to be fairly broad to be two-dimensional [37]. In narrow habitats (such as in [27]), some dependency of drift-induced expansion threshold on selection per loci would be expected [26]. Note that the apparent independence of the expansion threshold on *s*/r* does not imply that rate of range expansion should also be independent of the strength of selection.

**Fig 5:**
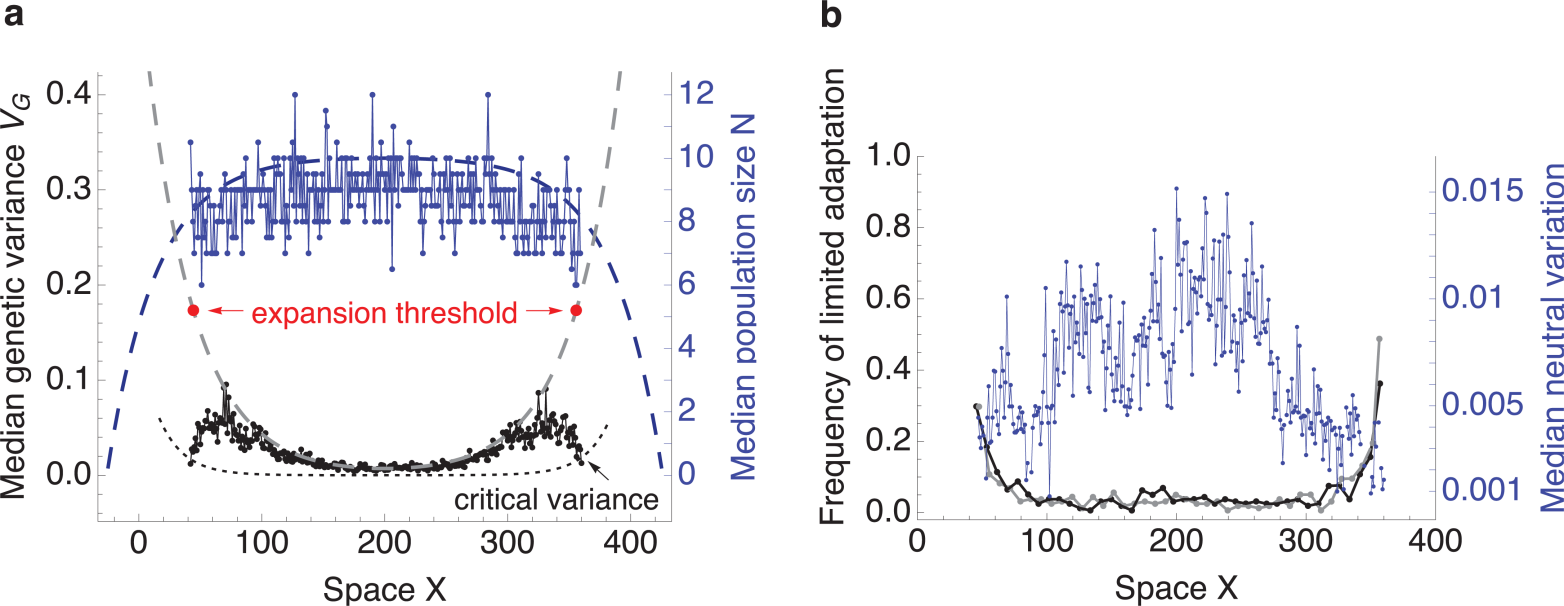
On a steepening environmental gradient, a sharp and stable range margin forms near the expansion threshold. This illustrative run shows that as the effective environmental gradient steepens away from the central location, adaptive genetic variance must increase correspondingly in order to maintain uniform adaptation. (**a**) Median population density stays fairly constant across the range (blue dots), following the deterministic prediction ($\hat{N}$, blue dashed line). Genetic variance (black dots) increases due to gene flow across the phenotypic gradient—the deterministic expectation is given by the grey dashed line (see Model section of Methods for details). Yet, as the environmental gradient steepens, genetic variance fails to increase fast enough, and near the expansion threshold, adaptation fails. The dotted line gives the corresponding critical genetic variance, below which only limited adaptation is expected in a phenotypic model with a fixed genetic variance ($A\approx B^{2}/2$, in which A is the standing genetic load; [24]). (**b**) As the environmental gradient steepens, the frequency of limited adaptation within the metapopulation increases (black and grey), and hence neutral variation decreases (blue). The black line gives the proportion of demes with limited adaptation after 50,000 generations, when the range margin appears stable; grey gives the proportion after 40,000 generations (depicted is an average over a sliding window of 15 demes). The median is given over the neutral spatial axis Y (with constant optimum); the trait mean, the population trait mean, variance, and population density in two-dimensional space is shown in S3 Fig, which also lists all the parameters.

In nature, conditions are unlikely to change uniformly. Abiotic environment (such as temperature, precipitation, solar radiation) does not, in general, change in a linear and concordant manner [38], and neither does the biotic environment, such as the pressure from competitors and predators, which affects the attainable population density and can increase the asymmetry in gene flow [39, 40]. I now investigate whether adaptation fails near the expansion threshold as conditions change across space. For example, we can imagine that the population starts well adapted in the central part of the available habitat, and as it expands, conditions become progressively more challenging (see S2A Fig); i.e., the effective environmental gradient *B* gets steeper. As the expanding population approaches the expansion threshold, adaptive genetic variance progressively decreases below the predicted value [13], which would be maintained by gene flow in the absence of genetic drift (Fig 5A, grey dashed line). This is a result of an increased frequency of demes with limited adaptation, leading to higher rates of extinctions and recolonisations, which reduce both adaptive and neutral diversity (see Fig 5B). Range expansion then ceases at the expansion threshold as the genetic variance drops to the critical value at which only limited adaptation is stable [24], assuming genetic variance is fixed (Fig 5A, dotted line). This is because although populations can persist with limited adaptation (Fig 4), the transient amount of genetic variance maintained under limited adaptation is almost never consistent with range expansion (see Fig 2, open circles). On a steepening gradient, a sharp and stable range margin forms. This contrasts to uniformly changing conditions (linear gradient, constant carrying capacity) in which populations steadily expand or contract.

**Fig 6:**
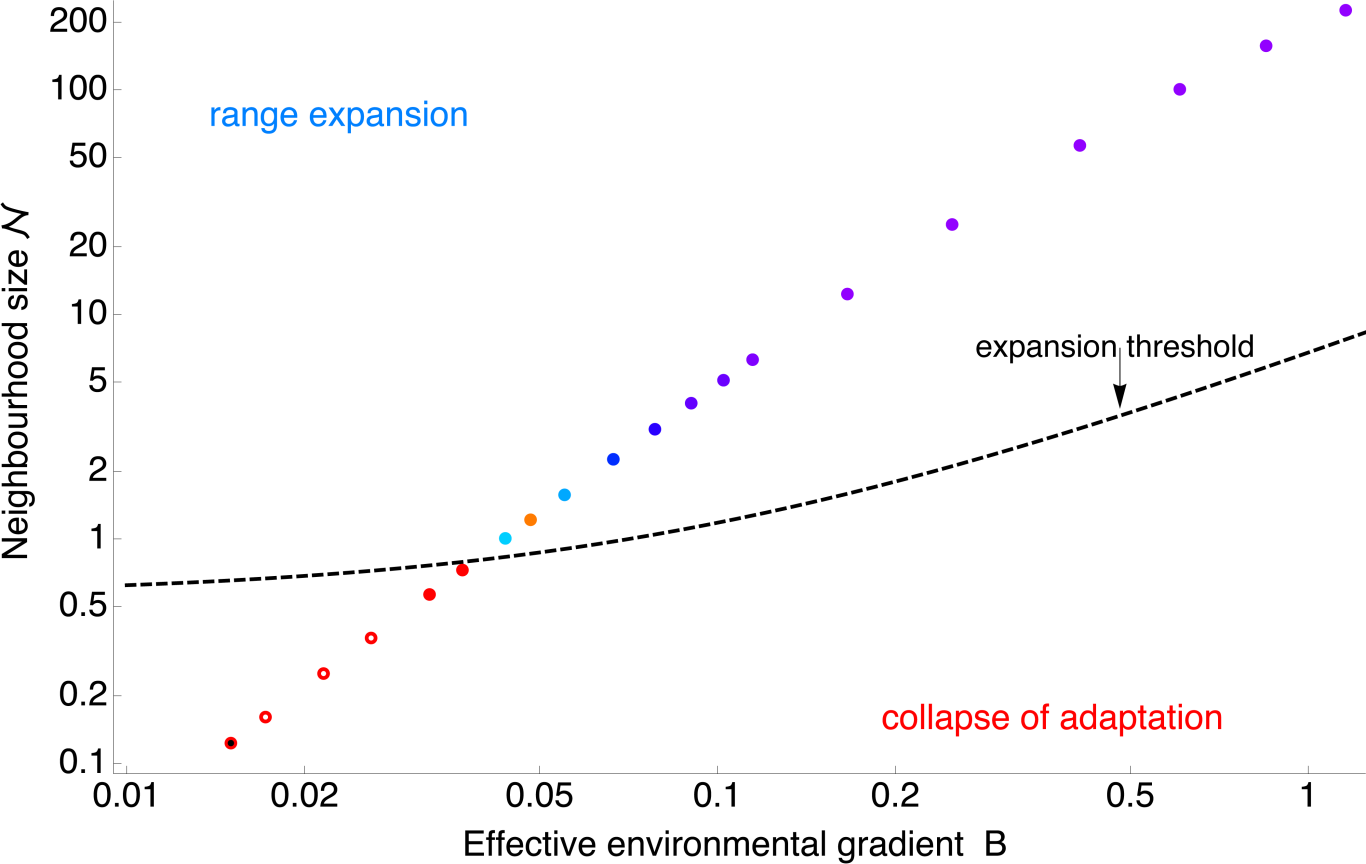
Dispersal aids adaptation in small populations because the neighbourhood size 𝒩 increases with the square of generational dispersal, whereas the effective environmental gradient *B* increases only linearly. This chart shows a set of simulated populations, with dispersal increasing from left to right and bottom to top. The hue of the dots indicates the rate of expansion (light to dark blue and purple) or collapse (orange to red). The rates of expansion and collapse are shown in dependency on *B* and 𝒩. Open circles indicate limited adaptation, in which a species’ range is fragmented and each subpopulation is only matching a single optimum, whilst its genetic variance is very small. As dispersal increases, population characteristics get above the expansion threshold (dashed line), and hence, uniform adaptation becomes stable throughout the species’ range. Local population density stays fairly constant, around *N* = 3.5, whilst total population size increases abruptly above the expansion threshold as the population maintains a wide range (not shown). Parameters for these simulations are given in the Individual-based simulations section of the Methods; the scaling of 𝒩 and *B* with dispersal σ is clear from the Methods, section Parameters. The rate of range change is not significantly different from zero for the first three simulations above the expansion threshold; black centre (bottom left) indicates extinction.

In a large population, the ability to adapt to heterogeneous environments is independent of dispersal: this is because both the local genetic variance (measured by standing genetic load), which enables adaptation to spatially variable environments, and the perceived steepness of the environmental gradient (measured by dispersal load) increase at the same rate with gene flow [13]. Yet, in small populations, dispersal is beneficial because the drift-reducing effect of dispersal overpowers its maladaptive effect. This is demonstrated in Fig 6—the neighbourhood size 𝒩 increases faster with dispersal than the effect of swamping by gene flow (*B*) does; hence, as dispersal increases, the population gets above the expansion threshold at which uniform adaptation can be sustained. Around the expansion threshold, a small change in dispersal (connectivity) can have an abrupt effect on adaptation across a species’ range and the species’ persistence. A small increase in dispersal can lead to recovery of uniform adaptation with an arbitrarily wide continuous range. Further increase of dispersal is merely enhancing the rate of range expansion at the expense of a slight cost to the mean fitness due to rising dispersal load and standing load and can be associated with further costs, such as Allee effect (see, e.g., [17]). Therefore, the expansion threshold provides an interpretation for optimality of an ‘intermediate’ dispersal, benefiting the species’ persistence.

## Discussion

Here, I have shown that adaptation fails when positive feedback between genetic drift, maladaptation, and population size reduces adaptive genetic variance to levels that are incompatible with continuous adaptation. The revealed expansion threshold differs qualitatively from the limit to adaptation identified previously [26] for a population living along a one-dimensional habitat. This is because in two dimensions, dispersal mitigates the loss of diversity due to genetic drift more effectively, such that it becomes (almost) independent of selection [34]. The expansion threshold implies that populations with very small neighbourhood sizes (𝒩 ⪅ 1/2), which suffer a severe reduction in neutral heterozygosity, will be prone to collapse based on demographic stochasticity alone. However, even in the absence of demographic stochasticity, genetic drift reduces the adaptive genetic variance required to sustain adaptation to a heterogeneous environment. The expansion threshold describes when this reduction due to genetic drift is incompatible with continuous adaptation, predicting a collapse of a species’ range. If the expansion threshold is reached as the species expands through its habitat, a sharp and stable range margin forms. If there is a drop below the expansion threshold throughout the species’ range, as after a sudden drop in carrying capacity, adaptation abruptly collapses throughout a species’ range. The result is either extinction or a fragmented metapopulation consisting of a conglomerate of subpopulations, each adapted to a single phenotypic optimum. It follows that near a range margin, we expect increased range fragmentation and a decrease in adaptive genetic variance. The threshold gives a theoretical base to the controversial issue of the importance of evolution (genetics) and ecology (demography) for assessing vulnerability of a species [41, 42]. The predicted sharp species’ range edge is in agreement with the reported lack of evidence for ‘abundant centre’ of a species’ range, which, although commonly assumed in macroecological theory, has little support in data [3, 11, 43, 44]. Lack of abundant centre is consistent both with uniform adaptation and with limited adaptation in a metapopulation.

The expansion threshold provides a general foundation to species-specific eco-evolutionary models of range dynamics [45]. Its components can be measured in wild populations, allowing us to test the robustness of the theory. First, the effective environmental gradient *B* can be measured as fitness loss associated with transplant experiments on a local scale, relative to a distance of generational dispersal along an environmental gradient. The environmental gradient can include both biotic and abiotic effects and their interactions [46]—notably, the effective environmental gradient *B* steepens due to increased asymmetry in gene flow when carrying capacity varies across space, e.g., because of partial overlap with competitors [40]. Second, the neighbourhood size 𝒩 can be estimated from neutral allele frequencies [47, 48]. Estimates of neighbourhood size are fairly robust to the distribution of dispersal distances [49]. Though near the expansion threshold, both the noisiness of the statistics and the homozygosity will increase due to local extinctions and recolonisations [50]. An alternative estimate of neighbourhood size can be also obtained from mark-recapture studies by measuring population density and dispersal (as an approximation for gene flow) independently [47].

Because the expansion threshold is free of any locus- or trait- specific measure, the result appears independent of genetic architecture, readily extending to multiple traits regardless of their correlations (compare to [51–55])—yet the mean fitness will decline because of ‘drift load’ as the number of traits independently optimised by selection increases [56, 57]. Hence, if the fitness landscape is highly complex, the expansion threshold constitutes a lower limit. Naturally, there can be further costs arising in a natural population that I have neglected here, such as the Allee effect [17]. In general, while the numerical constants may change when natural populations deviate in their biology from our model assumptions, the scale-free parameters identified in this study remain core drivers of the intrinsic dynamics within a species’ range. Notably, the early classic studies assuming fixed genetic variance [24] predicted that dispersal into peripheral populations is detrimental because it only inflates the effective environmental gradient *B*. Yet, when genetic variance can evolve, dispersal into small marginal populations also aids adaptation by increasing local genetic variance and by countering genetic drift. The net effect of dispersal into small marginal populations (below the expansion threshold) is then beneficial because their neighbourhood size increases faster with dispersal than the effective environmental gradient *B* steepens.

## Methods

### Model

I model evolution of a species’ range in a two-dimensional habitat, in which both population dynamics and evolution (in many additive loci) are considered jointly. The coupling is via the mean fitness $\bar{r}(\bar{z},N)$, which gives the growth rate of the population, and decreases with increasing maladaptation: $\bar{r}(\bar{z},N)=r_{e}(N)+{\bar{r}}_{g}(\bar{z})$. The ecological component of growth rate, $r_{e}$, can take various forms: here, the regulation is logistic so that fitness declines linearly with density *N*: *r*_e_ = *r*_m_(1−*N*/*K*), in which r_m_ is the maximum per capita growth rate in the limit of the local population density *N→0*. The carrying capacity *K* (for a perfectly adapted phenotype) is assumed uniform across space. The second term, $r_{g}(\bar{z})\leq 0$, is the reduction in growth rate due to deviation from the optimum. Selection is stabilising: the optimum *θ* changes smoothly with one spatial dimension (*x*): for any individual, the drop in fitness due to maladaptation is *r*_g_(*z*) = −(*z*−**θ*)*^*2*^/(2*V*_s_). Here, *V*_*s*_ gives the width of stabilising selection; strength of stabilising selection is *γ* = −*V*_*P*_/(2V_*s*_), in which *V*_*P*_ = *V*_*G*_+*V*_*E*_ is the phenotypic variance. A population with mean phenotype $\bar{z}$ has its fitness reduced by ${\bar{r}}_{g}(\bar{z})=-{\left(\bar{z}-\theta \right)}^{2}/(2V_{s})-V_{P}/(2V_{s})$. The phenotype z is determined by many di-allelic loci with allelic effects $\alpha_{i}$; the model is haploid, hence $\bar{z}=\sum_{i}\alpha_{i}p_{i}$, in which p_i_ is the allele frequency at locus *i*. Phenotypic variance is *V*_*P*_ = *V*_*G*_+*V*_*E*_. The loss of fitness due to environmental variance *V*_*E*_ can be included in $r_{m}^{*}=r_{m}-V_{E}/(2V_{s})$; *V*_*E*_ is a redundant parameter. Selection is ‘hard’: both the mean fitness (growth rate) and the attainable equilibrium density $\hat{N}=Kr^{*}/r_{m}=K(1-V_{G}/(2r_{m}V_{s}))$ decrease with maladaptation. Expected genetic variance maintained by gene flow in the absence of genetic drift is $V_{G}=b\sigma \sqrt{V_{s}}$ [13]; the contribution due to mutation is small, at mutation-section balance $V_{G,\mu /s}=2\mu V_{s}n_{l}$, in which *μ* gives the mutation rate per locus and *n*_*l*_ the number of loci.

### Individual-based simulations

Discrete-time individual-based simulations are set to correspond to the model with continuous time and space. The space is a two-dimensional lattice with spacing between demes of *δx* = 1. Every generation, each individual mates with a partner drawn from the same deme, with probability proportional to its fitness, to produce a number of offspring drawn from a Poisson distribution with mean of *Exp*[*r*(*z*, *N*)] (this includes zero). The effective diploid population density *N*_e_ hence equals half of the haploid population density *N*, and *𝒩* = 4*πN*_*e*_ *σ*^2^ = 2*πN*σ^2^. The life cycle is selection → mutation → recombination → birth → migration. Generations are nonoverlapping, and selfing is allowed at no cost. The genome is haploid with unlinked loci (the probability of recombination between any two loci is 1/2). The allelic effects *α*_i_ of the loci combine in an additive fashion; the allelic effects are uniform throughout this study, *α*_*i*_ ≡ *α*. Mutation is set to *μ* = 10^−6^, independently of the number of loci. Migration is diffusive with a Gaussian dispersal kernel. The tails of the dispersal kernel need to be truncated: truncation is set to two standard deviations of the dispersal kernel throughout, and dispersal probabilities and variance are adjusted so that the discretised dispersal kernel sums to 1 [58]. Simulations were run at the computer cluster of IST Austria using *Mathematica 9 (Wolfram)*. The code for the simulations, together with a working example, have been deposited as a single *.cdf file at Dryad Digital Repository, https://doi.org/10.5061/dryad.5vv37 [36]. This file can be viewed with *CDF Player*, a free application developed by *Wolfram Research*, and also contains all the figures with their underlying data.

#### Parameters

There are in total 10 parameters in the individual-based model, but only 7 are used to describe the model dynamics in continuous time. These are listed at the bottom of Table 1. They are the environmental gradient *b* = [0.012, 2], dispersal distance *σ=[0.1,1.3]*, carrying capacity for a well-adapted phenotype *K* = [3, 31], width of stabilising selection *V*_s=[0.005,6]_, the maximum intrinsic rate of increase *r*_*m*_ = [0.2, 2], and the mutation rate *μ*, fixed to *μ* = 10^−6^. The [min, max] interval gives the parameter range used in the 780 randomly sampled runs, with their distributions described in S3 Fig. The number of genes and demes is not included in the continuous time description (and hence the rescaling) because it assumes that space is not limiting and that all loci have equivalent effect with no statistical associations among them. In the individual-based model, the habitat width is set to be wide enough to be effectively two-dimensional under diffusive dispersal for thousands of generations [37]: 100 dispersal distances *σ* along the neutral direction and at least 10 cline (deterministic) widths along the gradient. The number of genes contributing to the adaptation across the species’ range is *n*_*l*_ = [5, 2996], with the estimated number of locally polymorphic genes between 1 and 299. Since mutation rate is fixed at *μ* = 10^−6^, the genomic mutation rate has a wide range, *U* = [5.10^−6^, 3.10^−3^], with median of *=* 10^−4^.

**Table 1:** Three scale-free parameters: B, 𝒩, and *s*/r* (top) describe the system. **T** Middle section gives informative derived parameters. The bottom section gives seven parameters of the model before rescaling, in which the seventh parameter, mutation rate *μ*, can be neglected because variance maintained by mutation-selection balance, *V*_G_, _*μ*/*s*_ *=* 2*μV*_s_*n*_*l*_, is typically much smaller than variance generated by gene flow across environments, $V_{G}=b\sigma \sqrt{V_{s}}$ The middle column gives the dimensions of the parameters.

param.	dim.	description
*B*	–	effective environmental gradient $B=b\sigma /(r^{*}\sqrt{2V_{s}})$
𝒩	–	neighbourhood size *N* = 4*πN*_*e*_ *σ*^2^ = 2*πN*σ^2^
*s*/r*	–	strength of selection per locus relative to the strength of density dependence
*s*	1/*T*	selection per locus: s ≡ *α*^2^/(2*V*_s_)
r*	1/*T*	rate of return to equilibrium pop. size: $r^{*}\equiv -N\partial \bar{r}/\partial N{|}_{N\to \hat{N}}=r_{m}-V_{G}/(2V_{s})$
*b*	*Z*/*D*	gradient in the environmental optimum
*V*_*s*_	*Z*^2^*T*	variance of stabilising selection
*σ*	$D/\sqrt{T}$	dispersal per generation
*K*	*T*/*D*^2^	max. carrying capacity (haploid) *K* = *N* when all phenotypes are perfectly adapted
*r*_*m*_	1/*T*	max. intrinsic rate of increase
*α*	Z	allelic effect
*μ*	1/T	mutation rate, *μ* ≡ 10^−6^

Abbreviations: D, distance; T, time; Z, trait

Parameters for Fig 3 are *b* = 0.18, *σ =* 0.52, *V*_s_ = 0.23, *K* = 26.7, *r*_*m*_ = 1 and *α* = 0.14, s = 0.04, *μ* = 10^−6^, 97 genes. Median genetic variance is at *V*_G_ = 0.031, deterministic prediction $V_{G}=b\sigma \sqrt{V_{s}}=0.45$ [13]. In Fig 4, the parameters are *b* = 1, *σ* = 0.4, *V*_*s*_ = 0.4, *K* = 4, *r*_*m*_= 1.2, and α = 0.1, s = 0.015, *μ* = 10^−6^, 874 genes. Median genetic variance within patches is around 0.02, whilst the maximum contribution by a single cline 1/4*α*^2^ = 0.0026; in contrast, variance maintained by gene flow under uniform adaptation [13] would be $V_{G}=b\sigma \sqrt{V_{s}}≐0.25$. Parameters for Fig 6 are *b*_0_ = 0.3, *σ* = [0.05, 3], *V*_s_ = 1, *K* = 4, $r_{m_{0}}=1$ and α = 0.05, *s* = 0.1, *μ* = 10^−6^, 1,000 genes, 1,000 demes along X, 200 demes along Y. These populations evolved for 500 generations.

### Continuous model

For any given additive genetic variance *V*_*G*_ (assuming a Gaussian distribution of breeding values), the change in the trait mean $\bar{z}$ over time satisfies:

$$\frac{\partial \bar{z}}{\partial t}=\frac{\sigma^{2}}{2}(\frac{\partial^{2}\bar{z}}{\partial x^{2}}+\frac{\partial^{2}\bar{z}}{\partial y^{2}})+\sigma^{2}(\frac{\partial^{2}\ln(N)}{\partial x}\frac{\partial \bar{z}}{\partial x}+\frac{\partial^{2}\ln(N)}{\partial y}\frac{\partial \bar{z}}{\partial y})+V_{G}\frac{\partial \bar{r}}{\partial \bar{z}}+\zeta .$$ (1)

The first term gives the change in the trait mean due to migration with mean displacement of *σ*; the second term describes the effect of the asymmetric flow from areas of higher density. The third term gives the change due to selection, given by the product of genetic variance and gradient in mean fitness [59, Eq 2]. The last term ζ gives the fluctuations in the trait variance due to genetic drift: $\zeta =\sqrt{V_{G,LE}/N}dW_{\zeta }(x,y,t)$, in which dW_*_ represents white noise in space and time [34, 60]. $V_{G,LE}=\sum_{i}\alpha_{i}^{2}p_{i}q_{i}$ denotes genetic variance assuming linkage equilibrium.

The trait mean is $\bar{z}=\sum_{i}\alpha_{i}p_{i}$ for a haploid model, in which *p*_*i*_ is the i-th allele frequency, *q*_i_ = 1−*p*_*i*_ and *α*_*i*_ is the effect of the allele on the trait—the change of the trait mean $\bar{z}$ as frequency of locus *i* changes from 0 to 1. For both haploid and diploid models, the allele frequencies *p*_*i*_ change as:

$$\frac{\partial p_{i}}{\partial t}=\frac{\sigma^{2}}{2}(\frac{\partial^{2}p_{i}}{\partial x^{2}}+\frac{\partial^{2}p_{i}}{\partial y^{2}})+\sigma^{2}(\frac{\partial p_{i}}{\partial x}\frac{\partial \ln(N)}{\partial x}+\frac{\partial p_{i}}{\partial y}\frac{\partial \ln(N)}{\partial y})+p_{i}q_{i}\frac{\partial \bar{r}}{\partial p_{i}}-\mu (p_{i}-q_{i})+\varepsilon .$$ (2)

The expected change of allele frequency due to a gradient in fitness and local heterozygosity is $p_{i}q_{i}\frac{\partial \bar{r}}{\partial p_{i}}=s_{i}p_{i}q_{i}(p_{i}-q_{i}-2\Delta_{i})$, in which selection at locus *i* is $s_{i}\equiv \alpha_{i}^{2}/(2V_{s})$ and $\Delta_{i}=(\bar{z}-bx)/\alpha_{i}$ [13, Appendix 3]. Here, the fourth term describes the change due to (symmetric) mutation at rate *μ*. The last term *ɛ* describes genetic drift [34, Eq 7]: $\varepsilon =\sqrt{\frac{p_{i}q_{i}}{N}}dW_{\varepsilon }(x,y,t)$, in which *N* is the haploid population density.

Population dynamics reflect diffusive migration in a two-dimensional habitat, growth due to the mean Malthusian fitness $\bar{r}$, and stochastic fluctuations. The number of offspring follows a Poisson distribution with mean and variance of *N*; fluctuations in population numbers are given by [61]: $\xi =\sqrt{N}dW_{\xi }(x,y,t)$:

$$\frac{\partial N}{\partial t}=\frac{\sigma^{2}}{2}(\frac{\partial^{2}N}{\partial x^{2}}+\frac{\partial^{2}N}{\partial y^{2}})+\bar{r}N+\xi$$ (3)

### Continuous model: Rescaling

The model can be simplified by rescaling [13, 59] time *t* relative to the strength of density dependence r*, distance *x* relative to dispersal *σ*, trait *z* relative to strength of stabilising selection $1/(2V_{s})$ and local population size *N* relative to equilibrium population size with perfect adaptation: $\hat{N}=Kr^{*}/r_{m}$, *T* = r*$t,X=x\sqrt{\frac{2r^{*}}{\sigma^{2}}},Z=\frac{z}{\sqrt{r^{*}V_{s}}},\tilde{N}=N/\hat{N}$. Note that near the equilibrium of a well-adapted population, $\tilde{N}\approx 1$.

The rescaled equations for evolution of allele frequencies and for demographic dynamics are

$$\begin{array}{c} \frac{\partial \tilde{N}}{\partial T}=\frac{\partial \tilde{N}}{\partial X^{2}}+\frac{\partial \tilde{N}}{\partial Y^{2}}+\bar{R}\tilde{N}+\sqrt{\frac{2\tilde{N}}{\hat{N}\sigma^{2}}}dW_{\tilde{\varsigma }\left(X,Y,T\right)}\\ \frac{\partial p_{i}}{\partial T}=\frac{\partial^{2}p_{i}}{\partial X^{2}}+\frac{\partial^{2}p_{i}}{\partial Y^{2}}+2\left(\frac{\partial p_{i}}{\partial X}\frac{\partial \text{ln}\left(\tilde{N}\right)}{\partial X}+\frac{\partial p_{i}}{\partial Y}\frac{\partial \text{ln}\left(\tilde{N}\right)}{\partial Y}\right)++\frac{s}{r^{*}}\left(p_{i}q_{i}-2\frac{\bar{Z}-BX}{\alpha^{*}}\right)-\frac{\mu }{r^{*}}\left(p_{i}-q_{i}\right)+\sqrt{\frac{p_{i}q_{i}}{\tilde{N}\hat{N}\sigma^{2}}}dW_{\tilde{\varepsilon }\left(X,Y,T\right)} \end{array}$$ (4)

in which $\overset{-}{R}\equiv \overset{-}{r}/r^{*}=1-\tilde{N}-{\left(BX-Z\right)}^{2}/2$.

The rescaled Eqs 4 show that four parameters fully describe the system. First, the effective environmental gradient, $B\equiv b\sigma /(r^{*}\sqrt{2V_{s}})$. Second, the strength of genetic drift $1/\hat{N}=1/\left(2\pi \hat{N}\sigma^{2}\right)$. The parameter $\hat{N}$ gives the neighbourhood size at an equilibrium with uniform adaptation. The third parameter is the strength of selection relative to the strength density dependence, *s*/r*; the scaled effect of a single substitution α* also scales with *s*/r*: $\alpha^{*}\equiv \alpha /\sqrt{r^{*}V_{s}}=\sqrt{2s/r^{*}}$. The effect of this third parameter *s*/r* is expected to be small, because typically $s\ll r^{*}$. Therefore, assuming throughout that *s* is uniform across loci is a reasonably justified simplification. The fourth parameter, *μ*/r*, will typically be very small and will be neglected throughout. Table 1 (top) summarises the full set that describes the system.

## Supporting information

S1 FigEffect on genetic drift on a cline width in two-dimensional habitats.**The figure shows how the cline width—and hence the local genetic variance—decrease with genetic drift in two-dimensional habitats. In contrast to linear habitats [62], the effect of drift is nearly independent of selection.** Genetic variance $V_{G}=\sum_{i}\alpha_{i}^{2}p_{i}q_{i}$ decreases as the clines steepen due to genetic drift. Fewer clines then contribute to genetic variance because the spacing between clines stays at α/b in order for the trait mean to match the optimum. The numerical approximation for the cline width under selection and genetic drift *w*_𝒩_ = *w*_*det*_ (1−10/*𝒩*)works well for weak genetic drift but breaks down for very small neighbourhood sizes (strong genetic drift, right). The deterministic cline width is given by $w_{det}=\frac{4\sigma }{\sqrt{2s}}$. Cline width for each locus is estimated from a central transect using the total heterozygosity across the whole habitat [62]: $w_{pq}=4\sum_{x=1}^{x=nd}p(x)q(x)\delta (x)$, in which δ(*x*) ≡ 1 gives the spacing of the spatial lattice and the lattice is *nd* demes wide. In principle, δ(*x*) can vary across space, as long as the sampling is dense enough around the cline centre. The position of the clines in a two-dimensional habitat is stabilised because the clines contribute to adaptation to a spatially variable trait under stabilising selection—the gradient in trait gives the direction for the transect. This figure represents a preliminary look at a complex problem that is a planned subject of a dedicated paper.(EPS)

S2 FigWith a steepening environmental gradient, a sharp range margin forms due to genetic drift.**The figure shows the population trait mean, variance, and population density in two-dimensional space.** (**a**) The gradient in trait mean follows the steepening environmental gradient, changing across one of the spatial axes, X (grey mesh). The red lines show the expansion threshold N≈6.3B+0.56. (**b**) As the environmental gradient steepens, local population density gradually declines (the grey mesh gives the predicted density). (**c**) As the environmental gradient steepens, so does the predicted genetic variance maintained by gene flow in the absence of genetic drift (grey mesh; $V_{G}=b\sigma \sqrt{V_{s}}$, [13]). Past the threshold, genetic variance starts to abruptly fall off the prediction, and adaptation begins to fail. The median values (across the neutral Y-space) are shown in Fig 5. Parameters: *σ* = 0.2, V_s_ = 0.5, *K*_0_ = 10, $r_{m_{0}}=1$ and *α* = 0.1, *s* = 0.01, *μ* = 10^−6^, 2,000 genes. The environmental gradient steepens away from the centre with *b*_0_ = 0.05 according to *b*(*x*) = 1/40 Exp(−1/40(*x*−200))+1/40 Exp(1/40(x−200)). The population was evolving for 50,000 generations, starting from a population adapted to the central part of the available habitat, about 100 demes wide.(EPS)

S3 FigDistribution of the parameters used in the 780 randomised simulation runs from Fig 2.Grey: all unscaled parameters used in the continuous time and space model. The unscaled parameters and their [min, max] ranges are environmental gradient *b* = [0.012, 2], dispersal distance σ=[0.1,1.3], carrying capacity for a well-adapted phenotype *K* = [3, 31], width of stabilising selection *V*_*s*_ = [0.005, 6], the maximum intrinsic rate of increase *r*_*m*_ = [0.2, 2]. White: the number of genes (an extra parameter in the individual-based model). The number of genes contributing to the adaptation across the species' range is *n*_*l*_ *=* [5, 2, 996], with the number of locally polymorphic genes between 1 and 299. Light blue: composite parameters: the strength of selection, s = *α*^2^ /(2*V*_*s*_) = [0.0002, 0.5], with median of *s* = 0.006. The effect size *α* = [0.01, 0.43]. Dark green: scale-free parameters: the effective environmental gradient *B* = [0.01, 1], with median *B* = 0.14 the neighbourhood size *𝒩* = [0.2, 60], with median 2.8; strength of selection relative to the strength of density dependence, *s*/r* = [0.00015, 1.34], with median *s*/r* = 0.006. Not pictured is the uniform mutation rate per locus (*μ* = 10^−6^), the number of demes along the two spatial directions, which was set to be at least 10 deterministic cline widths wide along the spatial axis with environmental gradient (X), whereas along the neutral spatial axis (Y), the habitat width was 100*σ*.(EPS)
